# Coffee Consumption and Risk of Breast Cancer: An Up-To-Date Meta-Analysis

**DOI:** 10.1371/journal.pone.0052681

**Published:** 2013-01-04

**Authors:** Xiu Juan Li, Zhao Jun Ren, Jian Wei Qin, Jian Hua Zhao, Jin Hai Tang, Ming Hua Ji, Jian Zhong Wu

**Affiliations:** 1 Department of Oncology, Xuzhou Medical College, Xuzhou, People's Republic of China; 2 Department of Pathology, Xuzhou Medical College, Xuzhou, People's Republic of China; 3 Department of General Surgery, Jiangsu Cancer Hospital, Nanjing, People's Republic of China; 4 Clinical Laboratory Center, Jiangsu Cancer Hospital, Nanjing, People's Republic of China; 5 Department of Radiation Oncology, Jiangsu Cancer Hospital, Nanjing, People's Republic of China; 6 Oncology of Central Laboratory, Jiangsu Cancer Hospital, Nanjing, People's Republic of China; King Faisal Specialist Hospital & Research Center, Saudi Arabia

## Abstract

**Objectives:**

This updated meta-analysis was conducted to assess the association between coffee consumption and breast cancer risk.

**Methods:**

We conducted a systematic search updated July 2012 to identify observational studies providing quantitative estimates for breast cancer risk in relation to coffee consumption. Pooled relative risks (RRs) with 95% confidence intervals (CIs) were calculated using a random-effects model, and generalized least square trend estimation was used to assess dose–response relationships.

**Results:**

A total of 26 studies (16 cohort and 10 case–control studies) on coffee intake with 49497 breast cancer cases were included in the meta-analysis. The pooled RR showed a borderline significant influence of highest coffee consumption (RR = 0.96; 95% CI 0.93–1.00), low-to moderate coffee consumption (RR = 0.99; 95% CI 0.95–1.04), or an increment of 2 cups/day of coffee consumption (RR = 0.98; 95% CI 0.97–1.00) on the risk of breast cancer. In stratified analysis, a significant inverse association was observed in ER-negative subgroup. However, no significant association was noted in the others.

**Conclusions:**

Our findings suggest that increased coffee intake is not associated with a significantly reduced risk of breast cancer, but we observe an inverse association in ER-negative subgroup analysis. More large studies are needed to determine subgroups to obtain more valuable data on coffee drinking and breast cancer risk.

## Introduction

Breast cancer is the most common type of female malignancy all over the world. Coffee, one of most known risk factors, may be crucial in the etiology of breast cancer [1]. The association between coffee intake and breast cancer risk is biologically plausible because of its complex make-up of chemicals, e.g., caffeine and polyphenolic compounds such as flavonoids and lignans [2]–[4]. Coffee can play a dual role as both a carcinogen, in which it enhances cell proliferation, and a chemo-preventive agent with anti-oxidative and weakly estrogenic properties [5], [6]. A number of previous epidemiologic studies have estimated the association between coffee consumption and breast cancer risk. However, the results were inconsistent. An earlier meta-analysis relating the consumption of coffee to cancer of various sites by Arab [7] reported a null association with breast cancer risk. But another meta-analysis published in 2009 suggested that high coffee consumption was associated with a borderline reduction of breast cancer risk [8]. Since the meta-analysis, several large prospective cohort studies have estimated the association between coffee consumption and breast cancer risk [9]–[16]. Therefore, to provide an updated results on this topic, we systematically conducted a meta-analysis by combining all available data of both case–control and cohort studies.

## Methods

### Search strategy

We searched the databases MEDLINE and EMBASE to identify relevant studies published in English through July 2012. The following keywords were used in searching: “caffeine”, “coffee”, or “dietary factors”, combined with “breast cancer”, “breast carcinoma,” or “breast neoplasm”. We also reviewed references cited in the selected articles. The eligible studies had to meet the following criteria: (i) They had a case–control or cohort study design; (ii) The outcome of interest was primary breast cancer; (iii) The exposure of interest was coffee consumption. (iv) Relative risk (RR) and their 95% confidence intervals (CIs) could be extracted or calculated from relevant articles.

### Data extraction

The following information was extracted from each included study: first author's last name, study design, country of origin, study period, number of cases and subjects, adjustment for potential confounders, the exposure to coffee consumption, RR and corresponding 95% CIs for every category of coffee intake. For each study, low coffee consumption was defined as the reference category, high coffee consumption as the greatest degree of control, and moderate coffee consumption fell in between. All the data were extracted independently by two authors (Li XJ and Ren ZJ), and the disagreement was solved by discussion.

### Statistical analysis

Study-specific RRs/odds ratios (ORs) and 95% CIs for low to moderate consumption and high consumption level were extracted from each study, and then we pooled the overall RR/OR using the inverse of the corresponding variances as weights. Because breast cancer is rare, ORs in case–control studies yield similar estimates of RR [17]. Heterogeneity of effect size across studies was tested by I^2^ statistics (I^2^>50% is considered significant). We calculated summary estimates of the RR using random-effects models, which consider both within- and between-study variation. Sensitivity analyses were also conducted, in which one study at a time was removed to analyze the influence of a single study on the pooled RR.

To obtain the information on a dose–response relationship, we considered the increment of 2 cup per day [18], [19]. For each study, we calculated the median cups of coffee consumption for each category by assigning the midpoint of upper and lower boundaries in each category as the average consumption. If the upper bound was not provided, we assumed that it had the same amplitude as the preceding category. Because this method requires the risk estimates with their variances for at least 3 quantitative exposure categories, we excluded studies showing two categories of exposure only [20]–[22]. And the summary RR for breast cancer risk with 2 cups/day increment of coffee consumption was obtained by pooling the corresponding study-specific RRs with random-effects models.

Studies were not eligible if the required data were not reported or could not be estimated. If coffee consumption was indicated by milliliter, we defined 125 ml of coffee equal to 1 cup.

The subgroup analyses according to geographic region, ER status, and menopausal status were performed to assess the potential effect of these variables on outcomes. The funnel plots with Begg's rank correlation and Egger regression tests were performed to detect publication bias [23]. All statistical analyses were performed with STATA (version 12.0; Stata Corp).

## Results

### Literature search

**Figure 1:**
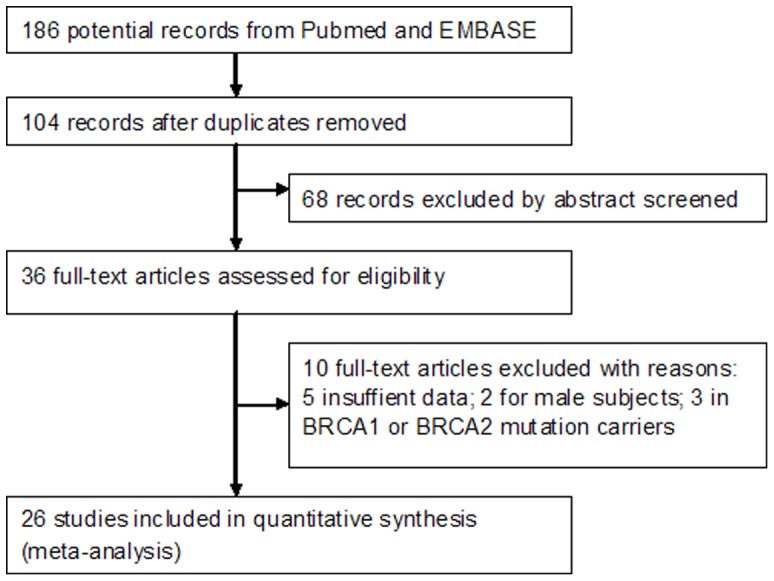
Flow chart of the selection of publications included in the meta-analysis.

We initially identified 186 potentially eligible studies. Most were excluded because the exposure or endpoint was not relevant to our analysis. After assessing the full-text of the 36 potentially relevant articles, we identified 26 eligible studies [9]–[16], [20]–[22], [24]–[38]. The main reasons for exclusion were as follows: 5 studies [39]–[43] did not provide a 95% CI. We tried to contact with authors for original data, but we got no reply. Because of relative small sample and poor study design, these original data was not necessary for meta-analysis; 2 studies [44], [45] were conducted among male subjects. We further excluded the other 3 studies, because they evaluated the association in BRCA1 or BRCA2 mutation carriers [46]–[48]. Finally, 16 cohort studies [10]–[16], [20], [24]–[31] and 10 case-control studies [9], [21], [22], [32]–[38] were included in the meta-analysis. A flow chart showing the study selection process is presented in Figure 1.

### Study characteristics

We identified 26 studies including 49497 incident cases of breast cancer and 863067 participants that were eligible for meta-analysis [9]–[16], [20]–[22], [24]–[38]. The characteristics of the included studies are summarized in Table S1. By study design, 10 case–control studies [9], [21], [22], [32]–[38] and 16 prospective cohort studies [10]–[16], [20], [24]–[31]. By geographic region, 12 studies were conducted in Europe [9], [12], [14], [16], [22], [24], [25], [28], [30], [34]–[36], 11 in the United States [10], [11], [13], [15], [20], [21], [26], [31], [32], [37], [38] and 3 in Asia [27], [29], [33]. One study [24] only adjusted for age, whereas the other 25 studies adjusted for a wide range of potential confounders for breast cancer, including age, BMI, family history of breast cancer, smoking, alcohol, geographic area, parity, age at first birth, age at menarche and menopause, oral contraceptive and other female hormone use.

### High and low to moderate coffee consumption

**Figure 2:**
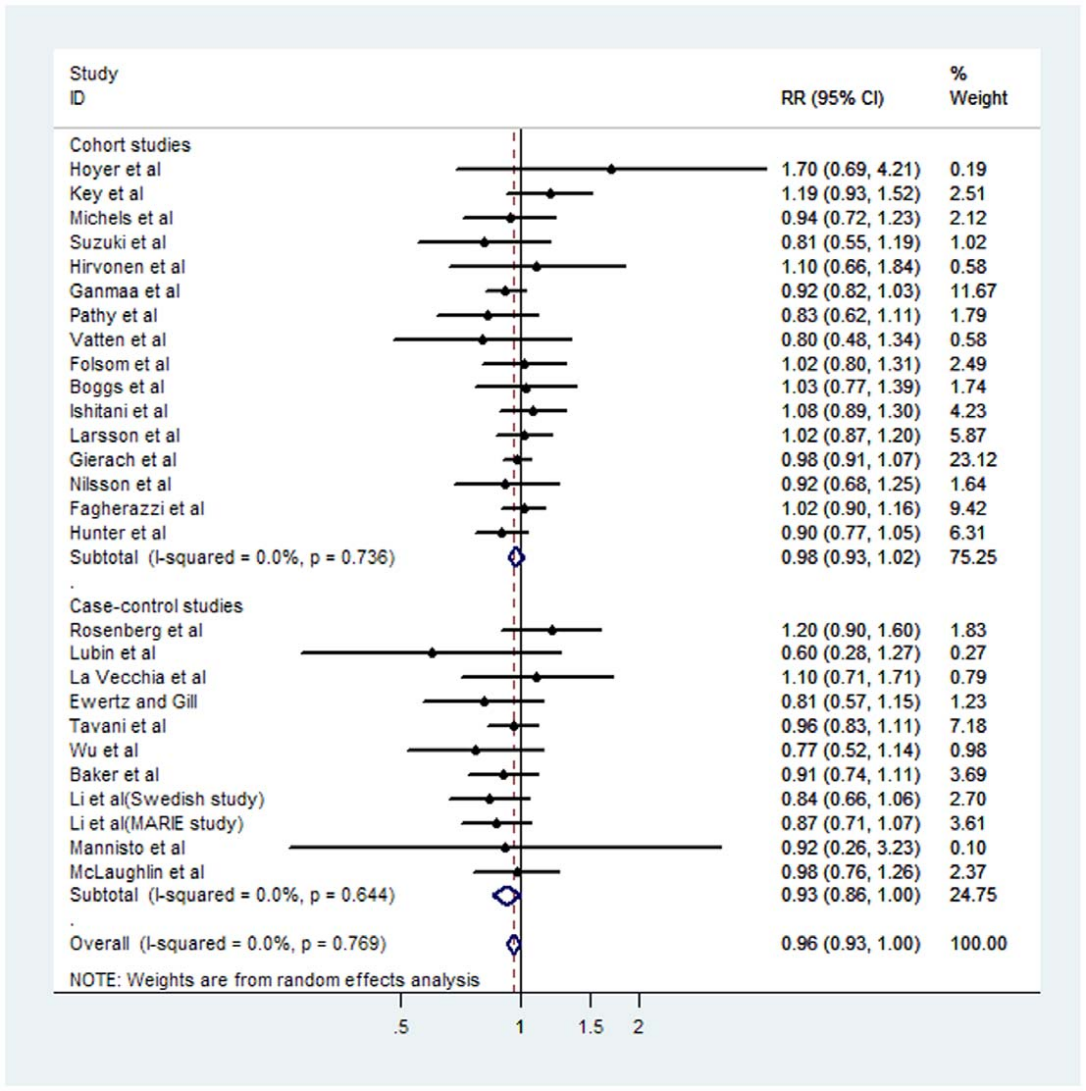
Forest plot of case–control and cohort studies of the risk of breast cancer for the highest versus lowest coffee drinking categories. The combined Relatives risks (RRs) and 95% confidence intervals (CIs) were calculated using the random-effects model.

**Figure 3:**
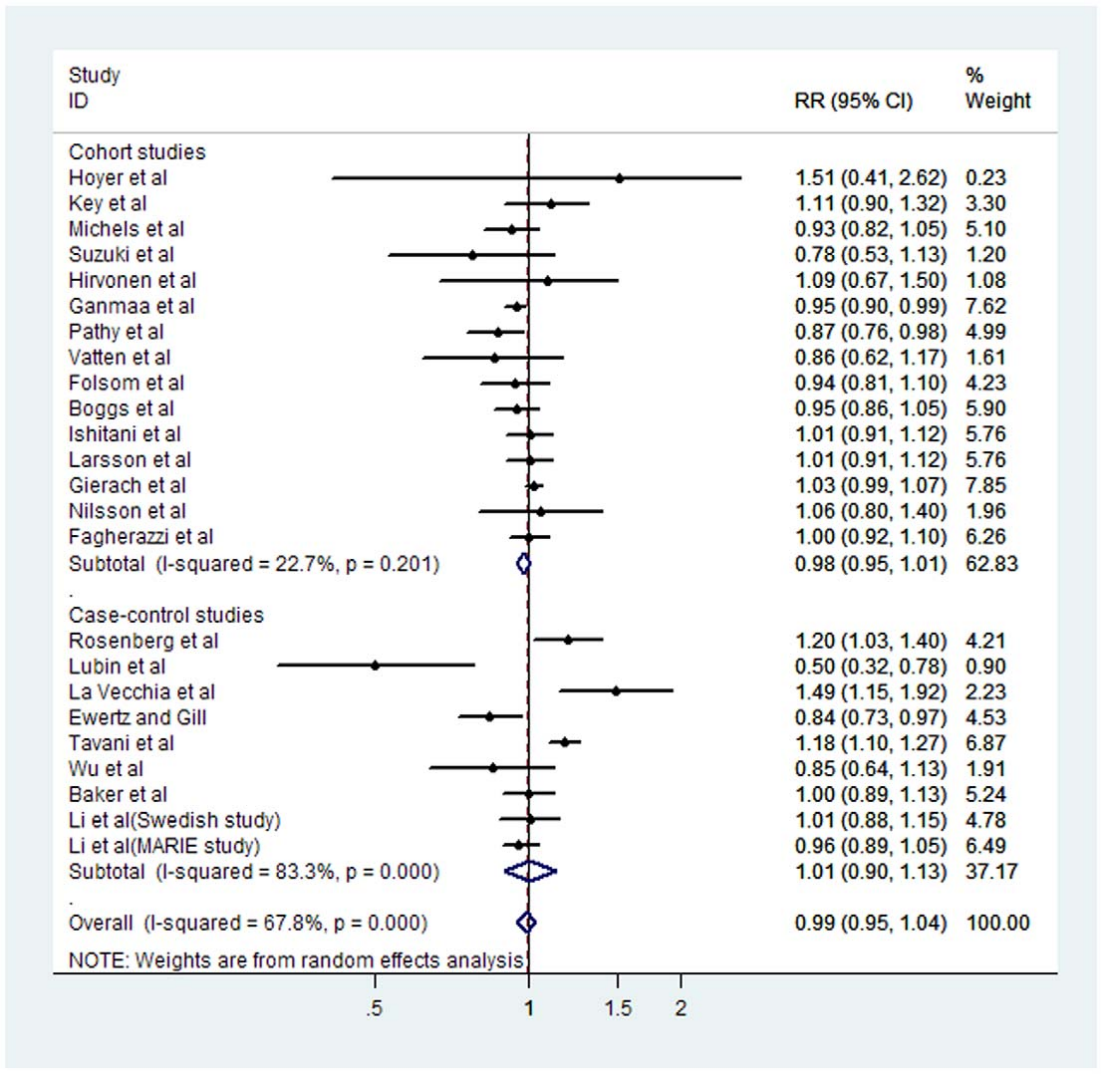
Forest plot of case–control and cohort studies of the risk of breast cancer for the low-to-moderate versus the lowest coffee drinking categories. The combined Relatives risks (RRs) and 95% confidence intervals (CIs) were calculated using the random-effects model.

The multivariable-adjusted RRs in each study and the pooled RR of breast cancer for the highest versus lowest categories of coffee intake are presented in Figure 2 and Table 1. The pooled RR of breast cancer for the highest versus lowest categories of coffee intake was 0.96 (95% CI 0.93–1.00). Stratifying by study design, the pooled RRs for case–control studies and cohort studies were 0.93 (95% CI 0.86–1.00) and 0.98 (95% CI 0.93–1.02), respectively. Stratifying by geographic region, the summary RRs were 0.96 (95% CI 0.90–1.02) for studies performed in Europe, 0.97 (95% CI 0.92–1.01) for studies performed in the United States, and 0.92 (95% CI 0.64–1.33) for studies performed in Asia. Stratifying by estrogen receptor(ER) status, the pooled RRs for ER-negative studies and ER-positive studies were 0.81 (95% CI 0.67–0.97) and 1.01 (95% CI 0.93–1.09), respectively. According to menopausal status, the pooled RR for premenopausal cancers was 1.00 (95% CI 0.72–1.40), and the pooled RR for postmenopausal cancers was 0.92 (95% CI 0.79–1.09). Figure 3 and Table 1 present the estimated RRs for low to moderate versus lowest coffee consumption, according to selected covariates. The summary RR was 0.99 (95% CI 0.95–1.04) for low to moderate versus lowest coffee consumption. In the subgroup analysis by study design, no increased risk was found for either cohort studies (OR = 0.98, 95% CI = 0.95–1.01) or case–control studies (OR = 0.98, 95% CI = 0.90–1.13). Stratified analyses were also performed according to geographic region. The RR was 1.00 (95% CI, 0.92–1.08) when considering 11 studies conducted in Europe, 0.78 (95% CI, 0.49–1.24) for 9 studies from United States and 1.00 (95% CI, 0.95–1.04) for 3 Asian studies. In addition, No significant differences by Menopause status were found.

**Table 1:** Main results of meta-analysis.

Group	No. of studies	Relative risk (95%CI)	Heterogeneity	
			I^2^	P-value
Highest vs. lowest				
All studies	26	0.96 (0.93–1.00)	0.0%	0.769
Study design				
All cohort studies	16	0.98 (0.93–1.02)	0.0%	0.736
All case-control studies	10	0.93 (0.86–1.00)	0.0%	0.644
Geographic area				
Europe	12	0.96 (0.90–1.02)	0.0%	0.780
United States	11	0.97 (0.92–1.01)	0.0%	0.703
Asia	3	0.92 (0.64–1.33)	58.1%	0.092
Estrogen receptor(ER) status				
ER-negative	5	0.81 (0.67–0.97)	26.1%	0.211
ER-positive	5	1.01 (0.93–1.09)	0.0%	0.909
Menopause status				
Premenopause	5	1.00 (0.72–1.40)	58.3%	0.048
Postmenopause	5	0.92 (0.79–1.09)	20.4%	0.285
Low-to-moderate vs. lowest				
All studies	23	0.99 (0.95–1.04)	67.8%	0.000
Study design				
All cohort studies	15	0.98 (0.95–1.01)	22.7%	0.201
All case-control studies	8	1.01 (0.90–1.13)	83.3%	0.000
Geographic area				
Europe	11	1.00 (0.92–1.08)	74.8%	0.000
United States	9	1.00 (0.95–1.04)	47.3%	0.056
Asia	3	0.98 (0.69–1.41)	82.7%	0.003
Menopause status				
Premenopause	2	0.94 (0.83–1.07)	0.0%	0.638
Postmenopause	2	0.99 (0.90–1.09)	0.0%	0.910
Increment of 2 cups/d				
All studies	23	0.98 (0.97–1.00)	0.0%	0.795
Study design				
All cohort studies	15	0.98 (0.97–1.00)	0.0%	0.554
All case-control studies	8	0.98 (0.96–1.00)	0.0%	0.802
Geographic area				
Europe	11	0.98 (0.97–1.00)	0.0%	0.920
United States	9	0.98 (0.96–1.01)	1.6%	0.421
Asia	3	0.98 (0.69–1.41)	49.1%	0.140
Menopause status				
Premenopause	2	0.91 (0.81–1.03)	44.7%	0.179
Postmenopause	2	0.93 (0.77–1.13)	86.0%	0.008

### Dose–response meta-analysis

**Figure 4:**
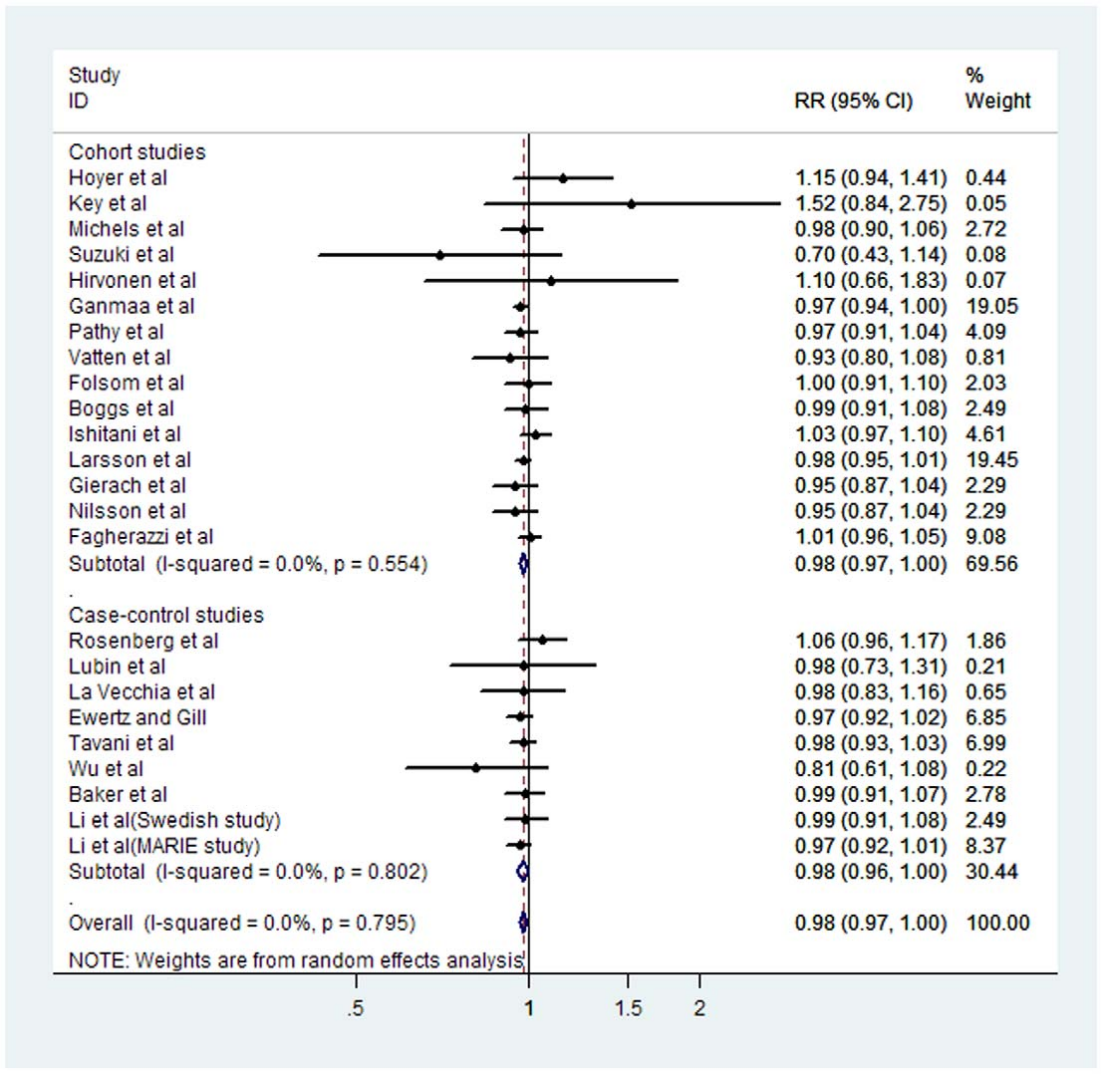
Forest plot of case–control and cohort studies of the risk of breast cancer for the increment of two cups of coffee per day. The combined Relatives risks (RRs) and 95% confidence intervals (CIs) were calculated using the random-effects model.

23 studies were included for the dose–response analysis of coffee intake and risk of breast cancer [9]–[16], [24]–[38]. The pooled RR for a 2 cups per day increment in coffee intake was 0.98 (95%CI 0.97–1.00) (Figure 4), similarly for cohort studies (pooled RR = 0.98, 95% CI = 0.97–1.00) and case–control studies (pooled RR = 0.98, 95% CI = 0.96–1.00) (Table 1). The summary RRs were 0.98 (95% CI 0.97–1.00) for studies conducted in Europe, 0.98 (95% CI 0.96–1.01) for studies conducted in the United States, and 0.98 (95% CI, 0.69–1.41) (Table 1) for studies conducted in Asia. When grouped by menopausal status, the pooled RR for premenopausal cancers was 0.91 (95% CI 0.81–1.03), and the pooled RR for postmenopausal cancers was 0.93 (95% CI 0.77–1.13) (Table 1).

### Publication bias

There was no indication of publication bias from either visualization of the funnel plot, Begg's test (p = 0.84), or Egger's test (p = 0.54). The sensitivity analysis confirmed the stability of our results.

## Discussion

This meta-analysis showed a borderline significant association between coffee consumption and decreased risk of breast cancer. The results of our meta-analysis were consistent with those in the earlier meta-analysis which contained 25250 cases [8]. Moreover, in the subgroup analysis by estrogen receptor(ER) status, we observed a significant inverse association between high coffee consumption and breast cancer risk. There was, however, relevant heterogeneity across studies, particularly according to study population and menopausal status. The relatively small sample size in Asia and the difference in the definition of menopausal status might contribute to the result.

The mechanisms by which coffee may affect breast carcinogenesis are complex and remain unclear. Coffee can both stimulate and suppress the development of mammary tumors in vitro [49], [50]. Coffee might contain compounds that differentially affect breast cancer of different ER subtypes. For example, coffee has been shown to significantly contribute to levels of plasma enterolactone [51], a phytoestrogen reported to be associated with a significant decrease in ER-negative breast cancer risk [52]. The presence of such compounds that specifically attenuates the risk of ER-negative breast cancer may contribute to our result in ER-negative subgroup analysis.

There were 5 studies [9], [10], [12], [13], [16] in our subgroup analysis by ER status, but a population-based case-control study reported by Li et al [9], accounted for our result of an inverse association between coffee intake and the risk of ER-negative breast cancer. In this multivariate-adjusted Swedish study, the pooled RR of ER-negative breast cancer for postmenopausal women who drank more than five cups of coffee per day was 0.43 (95% CI 0.25–0.72). However, in a large cohort study reported by Ishitani et al [11], there was a significant positive association between caffeine consumption and risk of developing estrogen receptor-negative and progesterone receptor-negative (ER−PR−) breast cancer according to hormone receptor status. Another cohort study [30], found no significant association between caffeine consumption and breast cancer risk according to hormone receptor status. Bageman et al [53], investigated the potential effect of CYP1A2 genotype on the relationship between coffee consumption and breast cancer risk among ER-negative patients. Although the CYP1A2 genotype alone did not affect breast cancer risk, the authors noted that, coffee consumption (≥2 cups per day) combined with the CYP1A2*1F A/A genotype is associated with a greater proportion of ER− tumors among patients with breast cancer in a population-based study (OR = 4.2; 95% CI, 1.9–9.3; *P* = 0.0002). Although the results appear inconsistent, it could be because of the different coffee-related variables measured. For instance, caffeine is only one out of the many different compounds contained in coffee, and thus caffeine intake is perhaps not a valid substitute for measuring the total effects of coffee consumption. In addition, the discrepancy might be attributed to other factors related to coffee drinking, such as brewing method, bean type, and caffeine content. Concerning relative small sample size, we cannot exclude the possibility that our findings in some subgroups may be a result of chance. More studies are needed to refute or confirm the associations that we observed in some subgroups.

The current meta-analysis had some advantages. First, the number of total cases included in the meta-analysis was substantial (n = 49497 cases). The pooled RRs of breast cancer for coffee intake were consistent with those in the previous meta-analysis which contained 25250 cases [8]. Second, we observed an inverse association between coffee intake and risk of ER-negative breast cancer. Breast cancer is characterized by genetic heterogeneity, encompassing different tumor types with distinct biologic features and clinical behaviors. So it indicated that researchers need to detail the clinical pathological parameters in future studies. Third, we found little evidence of publication bias in this meta-analysis.

Despite these advantages, some limitations of our meta-analysis should be acknowledged. First, misclassification of coffee intake and thus misclassified coffee components are inevitable due to self-reported intake. Second, a meta-analysis is unable to solve problems with confounding factors that could be inherent in the included studies. Third, although many of studies had adjusted for important risk factors for breast cancer, unmeasured variables may also have influenced results of individual studies. Fourth, not all studies were included for the dose–response analysis due to lack of data. Fifth, studies included in this meta-analysis were mostly conducted in Europe, the United States, and Asia. Therefore, additional research in other populations is warranted to generalize the findings. Sixth, potential publication bias might influence the findings, yet little evidence of publication bias was observed. Finally, the results of meta-analysis in some subgroups (e.g. estrogen receptor status and Menopause status) need further to be investigated, because of limited data.

In conclusion, our meta-analysis suggests that coffee consumption is not related to overall risk of breast cancer. However, our data suggest that high coffee consumption may decrease risk of ER–negative breast cancer, but these findings may be due to chance and warrant further study.

## Supporting Information

Checklist S1 A 27-item manuscript checklist.
(DOC)

Table S1 Summary characteristics of studies included in the meta-analysis.
(DOC)

## References

[1] Parkin MD, Bray F, Ferlay J, Pisani P (2005) Global Cancer Statistics, 2002. CA Cancer J Clin 55: 74–108. 15761078 10.3322/canjclin.55.2.74

[2] Scalbert A, Williamson G (2000) Dietary intake and bioavailability of polyphenols. J Nutr 130: 2073–2085. 10.1093/jn/130.8.2073S 10917926

[3] Allred KF, Yackley KM, Vanamala J, Allred CD (2009) Trigonelline is a novel phytoestrogen in coffee beans. J Nutr 139: 1833–1838. 19710155 10.3945/jn.109.108001

[4] Welsch CW, Scieszka KM, Senn ER, Dehoog JV (1983) Caffeine (1, 3, 7-trimethylxanthine), a temperate promoter of DMBA-induced rat mammary gland carcinogenesis. Int J Cancer 32 (4) 479–484. 6413433 10.1002/ijc.2910320415

[5] Olsen A, Knudsen KE, Thomsen BL, Loft S, Stripp C, et al (2004) Plasma enterolactone and breast cancer incidence by estrogen receptor status. Cancer Epidemiol Biomarkers Prev 13: 2084–2089. 15598765

[6] Lee WJ, Zhu BT (2006) Inhibition of DNA methylation by caffeic acid and chlorogenic acid, two common catechol-containing coffee polyphenols. Carcinogenesis 27: 269–277. 16081510 10.1093/carcin/bgi206

[7] Arab L (2010) Epidemiologic evidence on coffee and cancer. Nutr Cancer 62: 271–283. 20358464 10.1080/01635580903407122

[8] Tang N, Zhou B, Wang B, Yu R (2009) Coffee consumption and risk of breast cancer: a metaanalysis. Am J Obstet Gynecol 200: 290.e1–290.e9. 19114275 10.1016/j.ajog.2008.10.019

[9] Li J, Seibold P, Chang-Claude J, Flesch-Janys D, Liu J, et al (2011) Coffee consumption modifies risk of estrogen-receptor negative breast cancer. Breast Cancer Research 13 (3) R49. 21569535 10.1186/bcr2879 PMC3218935

[10] Boggs DA, Palmer JR, Stampfer MJ, Spiegelman D, Adams-Campbell LL, et al (2010) Tea and coffee intake in relation to risk of breast cancer in the Black Women's Health Study. Cancer Causes Control 21 (11) 1941–1948. 20680436 10.1007/s10552-010-9622-6 PMC3152948

[11] Ishitani K, Lin J, Manson JE, Buring JE, Zhang SM (2008) Caffeine Consumption and Risk of Breast Cancer in a Large Prospective Cohort of Women. Arch Intern Med 168 (18) 2022–2031. 18852405 10.1001/archinte.168.18.2022 PMC2574428

[12] Larsson SC, Bergkvist L, Wolk A (2009) Coffee and black tea consumption and risk of breast cancer by estrogen and progesterone receptor status in a Swedish cohort. Cancer Causes Control 20: 2039–2044. 19597749 10.1007/s10552-009-9396-x

[13] Gierach GL, Freedman ND, Andaya A, Hollenbeck AR, Park Y, et al (2012) Coffee intake and breast cancer risk in the NIH-AARP Diet and Health Study cohort. Int. J Cancer 131: 452–460. 10.1002/ijc.26372 PMC3290744 22020403

[14] Pathy NB, Peeters P, Van Gils C, Beulens JW, Van Der Graaf Y, et al (2010) Coffee and tea intake and risk of breast cancer. Breast Cancer Res Treat 121: 461–467. 19847643 10.1007/s10549-009-0583-y

[15] Nilsson LM, Johansson I, Lenner P, Lindahl B, Guelpen BV (2010) Consumption of filtered and boiled coffee and the risk of incident cancer: a prospective cohort study. Cancer Causes Control 21: 1533–1544. 20512657 10.1007/s10552-010-9582-x

[16] Fagherazzi G, Touillaud MS, Boutron-Ruault MC, Clavel-Chapelon F, Romieu I (2011) No association between coffee, tea or caffeine consumption and breast cancer risk in a prospective cohort study. Public Health Nutrition 14 (7) 1315–1320. 21466740 10.1017/S1368980011000371

[17] Bradburn MJ, Deeks JJ, Berlin JA, Russell Localio A (2007) Much ado about nothing: a comparison of the performance of meta-analytical methods with rare events. Stat Med 26 (1) 53–77. 16596572 10.1002/sim.2528

[18] Orsini N, Bellocco R, Greenland S (2006) Generalized least squares for trend estimation of sum-marized dose-response data. Stata J 6: 40–57.

[19] Greenland S, Longnecker MP (1992) Methods for trend estimation from summarized dose-response data, with applications to meta-analysis. Am J Epidemiol 135: 1301–9. 1626547 10.1093/oxfordjournals.aje.a116237

[20] Hunter DJ, Manson JE, Stampfer MJ (1992) A prospective study of caffeine, coffee, tea, and breast cancer (Abstract). Am J Epidemiol 136: 1000–1.

[21] McLaughlin CC, Mahoney MC, Nasca PC, Metzger BB, Baptiste MS, et al (1992) Breast cancer and methylxanthine consumption. Cancer Causes Control 3: 175–8. 1562707 10.1007/BF00051658

[22] Mannisto S, Pietinen P, Virtanen M, Kataja V, Uusitupa M (1999) Diet and the risk of breast cancer in a case-control study: does the threat of disease have an influence on recall bias? J Clin Epidemiol 52: 429–39. 10360338 10.1016/s0895-4356(99)00010-4

[23] Egger M, Davey Smith G, Schneider M, Minder C (1997) Bias in meta-analysis detected by a simple, graphical test. BMJ 315: 629–34. 9310563 10.1136/bmj.315.7109.629 PMC2127453

[24] Vatten LJ, Solvoll K, Løken EB (1990) Coffee consumption and the risk of breast cancer. A prospective study of 14,593 Norwegian women. Br J Cancer 62: 267–70. 2386741 10.1038/bjc.1990.274 PMC1971813

[25] Hoyer AP, Engholm G (1992) Serum lipids and breast cancer risk: a cohort study of 5,207 Danish women. Cancer Causes Control 3: 403–8. 1525320 10.1007/BF00051352

[26] Folsom AR, McKenzie DR, Bisgard KM, Kushi LH, Sellers TA (1993) No association between caffeine intake and postmenopausal breast cancer incidence in the Iowa Women's Health Study. Am J Epidemiol 138: 380–3. 8213743 10.1093/oxfordjournals.aje.a116870

[27] Key TJ, Sharp GB, Appleby PN, Beral V, Goodman MT, et al (1999) Soya foods and breast cancer risk: a prospective study in Hiroshima and Nagasaki, Japan. Br J Cancer 81: 1248–56. 10584890 10.1038/sj.bjc.6690837 PMC2374337

[28] Michels KB, Holmberg L, Bergkvist L, Wolk A (2002) Coffee, tea, and caffeine consumption and breast cancer incidence in a cohort of Swedish women. Ann Epidemiol 12: 21–6. 11750236 10.1016/s1047-2797(01)00238-1

[29] Suzuki Y, Tsubono Y, Nakaya N, Suzuki Y, Koizumi Y, et al (2004) Green tea and the risk of breast cancer: pooled analysis of two prospective studies in Japan. Br J Cancer 90: 1361–3. 15054454 10.1038/sj.bjc.6601652 PMC2409667

[30] Hirvonen T, Mennen LI, de Bree A, Castetbon K, Galan P, et al (2006) Consumption of antioxidant-rich beverages and risk for breast cancer in French women. Ann Epidemiol 16: 503–8. 16406814 10.1016/j.annepidem.2005.09.011

[31] Ganmaa D, Willett WC, Li TY, Feskanich D, Van Dam RM, et al (2008) Coffee, tea, caffeine and risk of breast cancer: a 22-year follow-up. Int J Cancer 122: 2071–6. 18183588 10.1002/ijc.23336 PMC4186696

[32] Rosenberg L, Miller DR, Helmrich SP, Kaufman DW, Schottenfeld D, et al (1985) Breast cancer and the consumption of coffee. Am J Epidemiol 122: 391–9. 4025289 10.1093/oxfordjournals.aje.a114120

[33] Lubin F, Ron E, Wax Y, Modan B (1985) Coffee and methylxanthines and breast cancer: a case-control study. J Natl Cancer Inst 74: 569–73. 3856060

[34] La Vecchia C, Talamini R, Decarli A, Franceschi S, Parazzini F, et al (1986) Coffee consumption and the risk of breast cancer. Surgery 100: 477–81. 3738766

[35] Ewertz M, Gill C (1990) Dietary factors and breast cancer risk in Denmark. Int J Cancer 46: 779–84. 2228305 10.1002/ijc.2910460505

[36] Tavani A, Pregnolato A, La Vecchia C, Favero A, Franceschi S (1998) Coffee consumption and the risk of breast cancer. Eur J Cancer Prev 7: 77–82. 9511854

[37] Wu AH, Yu MC, Tseng CC, Hankin J, Pike MC (2003) Green tea and risk of breast cancer in Asian Americans. Int J Cancer 106: 574–9. 12845655 10.1002/ijc.11259

[38] Baker JA, Beehler GP, Sawant AC, Jayaprakash V, McCann SE, et al (2006) Consumption of coffee, but not black tea, is associated with decreased risk of premenopausal breast cancer. J Nutr 136: 166–71. 16365077 10.1093/jn/136.1.166

[39] Le MG (1985) Coffee consumption, benign breast disease, and breast cancer. Am J Epidemiol 122: 721. 10.1093/oxfordjournals.aje.a114152 4025311

[40] Katsouyanni K, Trichopoulos D, Boyle P, Xirouchaki E, Trichopoulou A, et al (1986) Diet and breast cancer: a case-control study in Greece. Int J Cancer 38: 815–20. 3793261 10.1002/ijc.2910380606

[41] Jacobsen BK, Bjelke E, Kvåle G, Heuch I (1986) Coffee drinking, mortality, and cancer incidence: results from a Norwegian prospective study. J Natl Cancer Inst 76: 823–31. 3457969

[42] Levi F, La Vecchia C, Gulie C, Negri E (1993) Dietary factors and breast cancer risk in Vaud, Switzerland. Nutr Cancer 19: 327–35. 8346081 10.1080/01635589309514263

[43] Stensvold I, Jacobsen BK (1994) Coffee and cancer: a prospective study of 43,000 Norwegian men and women. Cancer Causes Control 5: 401–8. 7999961 10.1007/BF01694753

[44] Rosenblatt KA, Thomas DB, Jimenez LM, Fish B, McTierman A, et al (1999) The relationship between diet and breast cancer in men (United States). Cancer Causes Control 10: 107–13. 10231158 10.1023/a:1008808925665

[45] Johnson KC, Pan S, Mao Y (2002) Canadian Cancer Registries Epidemiology Research Group. Risk factors for male breast cancer in Canada, 1994–1998. Eur J Cancer Prev 11: 253–63. 12131659 10.1097/00008469-200206000-00009

[46] Gronwald J, Byrski T, Huzarski T, Cybulski C, Sun P, et al (2006) Influence of selected lifestyle factors on breast and ovarian cancer risk in BRCA1 mutation carriers from Poland. Breast Cancer Res Treat 95: 105–9. 16261399 10.1007/s10549-005-9051-5

[47] Nkondjock A, Ghadirian P, Kotsopoulos J, Lubinski J, Lynch H, et al (2006) Coffee consumption and breast cancer risk among BRCA1 and BRCA2 mutation carriers. Int J Cancer 118: 103–7. 16032702 10.1002/ijc.21296

[48] Kotsopoulos J, Ghadirian P, El-Sohemy A, Lynch HT, Snyder C, et al (2007) The CYP1A2 genotype modifies the association between coffee consumption and breast cancer risk among BRCA1 mutation carriers. Cancer Epidemiol Biomarkers Prev 16: 912–6. 17507615 10.1158/1055-9965.EPI-06-1074

[49] VanderPloeg LC, Welsch CW (1991) Inhibition by caffeine of ovarian hormone-induced mammary gland tumorigenesis in female GR mice. Cancer Lett 56: 245–50. 2021928 10.1016/0304-3835(91)90009-7

[50] Wolfrom D, Welsch CW (1990) Caffeine and the development of normal, benign and carcinomatous human breast tissues: a relationship? J Med 21: 225–50. 2079614

[51] Horner NK, Kristal AR, Prunty J, Skor HE, Potter JD, et al (2002) Dietary determinants of plasma enterolactone. Cancer Epidemiol Biomarkers Prev 11: 121–126. 11815409

[52] Olsen A, Knudsen KE, Thomsen BL, Loft S, Stripp C, et al (2004) Plasma enterolactone and breast cancer incidence by estrogen receptor status. Cancer Epidemiol Biomarkers Prev 13: 2084–2089. 15598765

[53] Bageman E, Ingvar C, Rose C, Jernstrom H (2008) Coffee Consumption and CYP1A2*1F Genotype Modify Age at Breast Cancer Diagnosis and Estrogen Receptor Status. Cancer Epidemiol Biomarkers Prev 17: 895–901. 18398030 10.1158/1055-9965.EPI-07-0555

