# Supplementary material for: Coffee Consumption and Risk of Breast Cancer: An Up-To-Date Meta-Analysis
Source: PLoS One. 2013 Jan 4;8(1):e52681. doi: 10.1371/journal.pone.0052681 (PMC3537715; doi:10.1371/journal.pone.0052681)
Supplement: Table S1 — Summary characteristics of studies included in the meta-analysis. (DOC) [file pone.0052681.s002.doc]

Table S1 Summary characteristics of studies included in the meta-analysis.

|  |  | Follow-up | Study | No.of | | Coffee | Relative risk |  |
| --- | --- | --- | --- | --- | --- | --- | --- | --- |
| Study | Country | period | design | cases | subjects | consumption | (95% CI) | Adjustments |
| Hoyer et al | Denmark | 1964-1986 | Cohort | 51 | 5,207 | 0-2 cups/d | 1.0(reference) | Social class, age at menarche, |
|  |  |  |  |  |  | 3-6 cups/d | 1.4(0.6-3.4) | menopause status, height, smoking status, |
|  |  |  |  |  |  | ≥7 cups/d | 1.7(0.7-4.3) | number of full-term pregnancies, |
|  |  |  |  |  |  |  |  | weight, BMI, alcohol consumption |
| Key et al | Japan | 1969-1993 | Cohort | 344 | 34,759 | ≤1 cup/wk | 1.00(reference) | Attained age, calendar period, |
|  |  |  |  |  |  | 2-4 cups/wk | 1.03(0.78-1.37) | city, age at time of bombing, |
|  |  |  |  |  |  | ≥5 cups/wk | 1.19(0.93-1.52) | radiation dose |
|  |  |  |  |  |  |  |  |  |
| Michels et al | Sweden | 1987-1997 | Cohort | 1271 | 59,036 | ≤1 cup/wk | 1.00(reference) | Age, family history of breast cancer, |
|  |  |  |  |  |  | 2-4 cups/wk | 0.81(0.54-1.22) | height, BMI, education, parity, |
|  |  |  |  |  |  | 1 cup/d | 0.99(0.75-1.28) | age at first birth, alcohol consumption, |
|  |  |  |  |  |  | 2-3 cups/d | 0.94(0.79-1.12) | total caloric intake |
|  |  |  |  |  |  | ≥4 cups/d | 0.94(0.75-1.28) |  |
| Suzuki et al | Japan | 1984-1997 | Cohort | 222 | 35,004 | Never | 1.00(reference) | Age, types of health insurance, |
|  |  |  |  |  |  | Occasionally | 0.78(0.53-1.13) | age at menarche, menopausal status, |
|  |  |  |  |  |  | ≥1 cups/d | 0.81(0.55-1.18) | age at first birth, parity, BMI, smoking, |
|  |  |  |  |  |  |  |  | mother's history of breast cancer, alcohol |
|  |  |  |  |  |  |  |  | drinking, tea consumption frequencies |
| Hirvonen et al | France | 1994-2002 | Cohort | 95 | 4,396 | 0-111 mL/d | 1.00(reference) | Age, smoking, number of children, |
|  |  |  |  |  |  | 112-252 mL/d^a^ | 1.07(0.64-1.79) | use of oral contraception, menopausal |
|  |  |  |  |  |  | ≥253 mL/d^a^ | 1.10(0.66-1.84) | status, family history of breast cancer, |
| Ganmaa et al | United | 1980-2002 | Cohort | 5272 | 85,987 | ＜1 cup/mo | 1.00(reference) | Age, BMI, physical activity, smoking status, |
|  | States |  |  |  |  | 1 cup/mo | 1.01(0.92-1.12) | height, alcohol intake, tea intake, |
|  |  |  |  |  |  | 4.9 cups/wk |  | age at menarche and menopause, |
|  |  |  |  |  |  | 5 cups/wk- | 0.92(0.84-1.01) | menopausal status, use of hormone therapy, |
|  |  |  |  |  |  | 1.9 cups/d |  | parity age at first birth, weight change, |
|  |  |  |  |  |  | 2-3.9 cups/d | 0.93(0.85-1.02) | duration of postmenopausal hormone use, |
|  |  |  |  |  |  | ≥4 cups/d | 0.92(0.82-1.03) | family history of breast cancer |
|  |  |  |  |  |  |  |  |  |
| Pathy et al | Netherlands | 1993-2007 | Cohort | 681 | 27,323 | 0 cup/d | 0.74(0.52-1.05) | Age, smoking status, educational status, |
|  |  |  |  |  |  | 0.1-1.0 cups/d | 1.00(reference) | alcohol intake, energy intake, BMI, |
|  |  |  |  |  |  | 1.1-2.0 cups/d | 0.93(0.74-1.17) | energy adjusted fiber and saturated |
|  |  |  |  |  |  | 2.1-3.0 cups/d | 0.90(0.68-1.19) | fat intake, ever use of oral contraceptives, |
|  |  |  |  |  |  | 3.1-5.0 cups/d | 0.83(0.65-1.06) | physical activity level, age at menarche, |
|  |  |  |  |  |  | ＞5 cups/d | 0.83(0.62-1.11) | presence of hypercholesterolemia, parity, |
|  |  |  |  |  |  |  |  | family history of breast cancer, cohort |
| Vatten et al | Norway | 1974-1985 | Cohort | 152 | 14593 | ≤2cups/d | 1.00(reference) | Age |
|  |  |  |  |  |  | 3-4 cups/d | 0.9(0.6-1.4) |  |
|  |  |  |  |  |  | 5-6 cups/d | 0.8(0.5-1.3) |  |
|  |  |  |  |  |  | ≥7 cups/d | 0.8(0.5-1.4) |  |
| Hunter et al | United States | 1980-1987 | Cohort | 1439 | 89494 | ＜1 cup/mo | 1.00(reference) | Established breast cancer risk factors |
|  |  |  |  |  |  | ＞4 cups/d | 0.90(0.76-1.04) |  |
| Folsom et al | United States | 1986-1990 | Cohort | 580 | 41837 | Never or ＜1 cup/mo | 1.00(reference) | Age, waist/hip ratio, no.of live births, age at first live birth, age at menarche, family history Of breast cancer, |
|  |  |  |  |  |  | 1 cup/mo-4 cups/wk | 0.87(0.66-1.14) | family history×waist/hip ratio, family history×no.of live births |
|  |  |  |  |  |  | 5-7 cups/wk | 0.96(0.73-1.27) |  |
|  |  |  |  |  |  | 2-3 cups/d | 0.98(0.78-1.23) |  |
|  |  |  |  |  |  | ≥4 cups/d | 1.02(0.79-1.30) |  |
| Rosenberg et al | United States | 1975-1982 | HCC | 2642 | 4124 | 0 | 1.00(reference) | Age, race, religion, cigarette smoking, |
|  |  |  |  |  |  | 1-2 cups/d | 1.2(1.0-1.5) | age at menarche, age at first pregnancy, |
|  |  |  |  |  |  | 3-4 cups/d | 1.2(1.0-1.6) | parity, type of menopause, age at |
|  |  |  |  |  |  | ≥5 cups/d | 1.2(0.9-1.6) | menopause, history of breast cancer, |
|  |  |  |  |  |  |  |  | BMI based on weight, at time of |
|  |  |  |  |  |  |  |  | interview, years of education, tea |
|  |  |  |  |  |  |  |  | consumption, alcohol consumption, |
|  |  |  |  |  |  |  |  | location, of the hospital, year of |
|  |  |  |  |  |  |  |  | interview, number of previous |
|  |  |  |  |  |  |  |  | nonobstetric hospitalizations. |
| Lubin et al | Israel | 1975-1978 | PCC | 807 | 1614 | ≤1 cup/wk | 1.00(reference) | Hormonal and nutritional factors |
|  |  |  |  |  |  | 1 cup/wk-1 cup/d | 0.5(0.3-0.9) |  |
|  |  |  |  |  |  | 2-3 cups/d | 0.5(0.2-0.9) |  |
|  |  |  |  |  |  | ≥4 cups/d | 0.6(0.2-0.9) |  |
| La Vecchia et al | Italy | 1980-1985 | HCC | 616 | 1232 | 0 | 1.00(reference) | Age, geographic area, parity, age at first birth, |
|  |  |  |  |  |  | ＜2 cups/d | 1.6(1.1-2.4) | age at menarche and menopause, oral |
|  |  |  |  |  |  | 2-3 cups/d | 1.4(1.0-2.0) | contraceptive and other female hormone use, |
|  |  |  |  |  |  | ≥4 cups/d | 1.1(0.7-1.7) | cigarette smoking, alcohol consumption |
| Ewertz and Gill | Denmark | 1983-1984 | PCC | 1431 | 2720 | ≤2 cups/d | 1.00(reference) | Age at diagnosis and place of residence |
|  |  |  |  |  |  | 3-5 cups/d | 0.83(0.68-1.00) |  |
|  |  |  |  |  |  | 6-9 cups/d | 0.86(0.69-1.07) |  |
|  |  |  |  |  |  | ≥10 cups/d | 0.81(0.57-1.15) |  |
| McLaughlin et | United States | 1982-1984 | PCC | 1617 | 3234 | Never | 1.00(reference) |  |
| al |  |  |  |  |  | Ever | 0.98(0.76-1.26) | Age, country of residence, race, menstrual |
|  |  |  |  |  |  |  |  | status, age at first live birth, diagnosis of |
|  |  |  |  |  |  |  |  | benign breast disease, family history of breast |
|  |  |  |  |  |  |  |  | cancer, alcohol consumption |
| Tavani et al | Italy | 1983-1994 | HCC | 5968 | 11446 | No drinkers | 1.00(reference) | Study/center, age, education, BMI, smoking |
|  |  |  |  |  |  | ＞0 to ＜2 cups/d | 1.17(1.03-1.33) | status, total alcohol intake, age at menarche and menopause, parity and age at first birth, |
|  |  |  |  |  |  | 2 cups/d | 1.17(1.04-1.33) | use of oral contraceptive, use of hormone |
|  |  |  |  |  |  | ＞2 to ＜4 cups/d | 1.21(1.06-1.37) | replacement therapy, history of breast  disease, family history of breast cancer |
|  |  |  |  |  |  | ≥4 cups/d | 0.96(0.83-1.11) |  |
| Mannisto et al | Finland | 1990-1995 | PCC | 310 | 764 | ＜120 g/d | 1.00(refrence) | Age, area, age at menarche, age at first |
|  |  |  |  |  |  | ＞488 g/d | 0.92(0.26-3.21)^b^ | full-term pregnancy, use of oral |
|  |  |  |  |  |  |  |  | contraceptives, use of estrogen replacement therapy, first-degree family history of breast cancer, history of benign breast disease, level of education, alcohol intake, smoking, leisure activity, waist-to-hip ratio |
| Wu et al | United States | 1995-1998 | PCC | 501 | 1094 | 0 | 1.00(reference) | Age, Asian ethnicity, birthplace, education, |
|  |  |  |  |  |  | ＞0-120 mL/d^a^ | 0.91(0.60-1.38) | age at menarche, pregnancy, current BMI, |
|  |  |  |  |  |  | ＞120 to ≤240 mL/d^a^ | 0.80(0.55-1.19) | total caloric intake, menopausal status, use of  menopausal hormones, intake of soy and dark |
|  |  |  |  |  |  | ＞240 mL/d^a^ | 0.77(0.52-1.13) | green vegetables, smoking history, alcohol |
|  |  |  |  |  |  |  |  | intake, physical activity, family history of  breast cancer |
| Baker et al | United States | 1982-1998 | HCC | 1932 | 3827 | 0 | 1.00(reference) | Age, residence, age at birth of first child |
|  |  |  |  |  |  | ＜1 cup/d | 0.95(0.75-1.19)^b^ |  |
|  |  |  |  |  |  | 1 cup/d | 0.93(0.74-1.17)^b^ |  |
|  |  |  |  |  |  | 2-3 cups/d | 1.07(0.91-1.27)^b^ |  |
|  |  |  |  |  |  | ≥4 cups/d | 0.91(0.74-1.11)^b^ |  |
| Li et al | Sweden | 1993-1995 | PCC | 2818 | 5929 | ≤1 cup/d | 1.00(reference) | Age, potential confounders (hormone smoked |
|  |  |  |  |  |  | ＞1 to 3 cups/d | 1.01(0.84-1.23) | replacement therapy (HRT), ever/never, ever |
|  |  |  |  |  |  | ＞3 to 5 cups/d | 1.00(0.82-1.22) | > 1 y or >100 cigarettes, and education |
|  |  |  |  |  |  | ＞5 cups/d | 0.84(0.66-1.06) | (elementary school, junior secondary school, |
|  |  |  |  |  |  |  |  | high school or university)) and average daily |
|  |  |  |  |  |  |  |  | alcohol consumption (g/day). |
|  | Germany | 2003-2005 | PCC | 2651 | 8046 | ≤1 cup/d | 1.00(reference) | Age and study region potential confounders: |
|  |  |  |  |  |  | ＞1 to 3 cups/d | 0.97(0.87-1.07) | hormone replacement therapy (HRT, |
|  |  |  |  |  |  | ＞3 to 5 cups/d | 0.95(0.82-1.10) | ever/never), ever smoked > 100 cigarettes, and |
|  |  |  |  |  |  | ＞5 cups/d | 0.87(0.71-1.07) | education (low, medium, high) and average |
|  |  |  |  |  |  |  |  | daily alcohol consumption (continuous, in g/d) |
| Boggs et al | African-American | 1995-2007 | Cohort | 1268 | 52062 | Never, ＜1 cup/mo | 1.00(reference) | Age, energy intake, age at menarche, BMI at age 18, family history of breast cancer, first |
|  |  |  |  |  |  | ＜1 cup/d | 0.98(0.85-1.12) | education, geographic region, parity, age at |
|  |  |  |  |  |  | 1 cup/d | 0.91(0.76-1.09) | birth, oral contraceptive use, menopausal |
|  |  |  |  |  |  | 2-3 cups/d | 0.94(0.77-1.15) | status, age at menopause, female hormone use, |
|  |  |  |  |  |  | ≥4 cups/d | 1.03(0.77-1.39) | vigorous activity, smoking status, and alcohol |
|  |  |  |  |  |  |  |  | intake |
| Ishitani et al | United States | 1992-2004 | Cohort | 1188 | 38432 | Almost never | 1.00(reference) | Age, randomized treatment assignment, body |
|  |  |  |  |  |  | ＜1 cup/d | 0.97(0.79-1.18) | mass index, physical activity, total energy |
|  |  |  |  |  |  | 1 cup/d | 0.98(0.81-1.19) | intake, alcohol intake, multivitamin use, age at |
|  |  |  |  |  |  | 2-3 cups/d | 1.05(0.89-1.22) | menopause, age at menarche, age at first |
|  |  |  |  |  |  | ≥4 cups/d | 1.08(0.89-1.30) | pregnancy lasting ≥6months, number of |
|  |  |  |  |  |  |  |  | pregnancies lasting ≥6months, menopausal status, postmenopausal hormone use, prior hysterectomy, prior bilateral oophorectomy, smoking status, family history of breast cancer in mother or a sister, and history of benign  breast disease |
| Larsson et al | Sweden | 1987-2007 | Cohort | 2952 | 61433 | ＜1 cup/d | 1.00(reference) | Age, education (primary school, high school, |
|  |  |  |  |  |  | 1 cup/d | 1.05(0.90-1.23) | university), body mass index (＜18.5, |
|  |  |  |  |  |  | 2-3 cups/d | 0.97(0.84-1.11) | 18.5–24.9, 25–29.9, ≥30 kg/m 2), height (in |
|  |  |  |  |  |  | ≥4 cups/d | 1.02(0.87-1.20) | cm; continuous), parity (nulliparous, 1–2, ≥ |
|  |  |  |  |  |  |  |  | 3), age at first birth (nulliparous, ＜26, 26–30, ≥31 years), age at menarche (≤12, 13, ≥14 years), age at menopause (＜51, ≥51 years), use of oral contraceptives (ever/never), use of postmenopausal hormones (ever/never), family history of breast cancer (yes/no), and intakes of total energy (kcal/day; continuous), alcohol (nondrinkers, ＜3.4, 3.4–9.9, ≥10.0 g/day) and tea (never/seldom, ＜1 cup/day, 1 cup/day, ≥2 cups/day) |
| Gierach et al | United States | 1995-2006 | Cohort | 9915 | 198404 | Never | 1.00(reference) | Age (continuous), race/ethnicity, education, |
|  |  |  |  |  |  | ≤2 cups/wk | 1.06(0.97-1.15) | BMI (kg/m2), smoking status and dose, |
|  |  |  |  |  |  | 3-6 cup/wk | 1.00(0.91-1.10) | alcohol, proportion of total energy from fat |
|  |  |  |  |  |  | 1 cup/d | 1.02(0.94-1.09) | (quintiles), age at first live birth, menopausal |
|  |  |  |  |  |  | 2-3 cups/d | 1.02(0.95-1.09) | hormone therapy use, history of breast biopsy |
|  |  |  |  |  |  | ≥4 cups/d | 0.98(0.91-1.07) | and family history of breast cancer. |
| Nilsson et al | United States | 1992-2007 | Cohort | 587 | 30639 | ＜1 occasion/d | 1.00(reference) | Age, sex, BMI, smoking, education, and |
|  |  |  |  |  |  | 1-3 occasions/d | 1.06(0.80-1.40) | recreational physical activity |
|  |  |  |  |  |  | ≥4 occasions/d | 0.92(0.68-1.25) |  |
| Fagherazzi et al | France | 1993-2005 | Cohort | 2868 | 67703 | Non-consumers | 1.00(reference) | Baseline variables (total energy intake, ever |
|  |  |  |  |  |  | ≤1 cup/d | 1.02(0.91-1.15) | use of oral contraceptives, age at menarche, |
|  |  |  |  |  |  | 1.1-3 cups/d | 0.98(0.85-1.11) | age at menopause, number of children, age at |
|  |  |  |  |  |  | ＞3 cups/d | 1.02(0.90-1.16) | first pregnancy, history of breast cancer in the |
|  |  |  |  |  |  |  |  | family and years of schooling) and time-dependent variables (current use of postmenopausal hormone therapy (for postmenopausal women only), personal history of benign breast disease, menopausal status and BMI). |

HCC, hospital-based case-control study; PCC, population-based case-control study.

^a^ One cup of coffee was considered equal to about 125 mL; ^b^ We calculated a single RR (95% CI) estimate for total subjects by means of a weighted average of premenopausal RR (95% CI) and postmenopausal RR (95% CI).
